# Sleep Health and White Matter Integrity in the UK Biobank

**DOI:** 10.1111/jsr.70034

**Published:** 2025-03-12

**Authors:** Roxana Petri, Florian Holub, Julian E. Schiel, Bernd Feige, Martin K. Rutter, Sandra Tamm, Dieter Riemann, Simon D. Kyle, Kai Spiegelhalder

**Affiliations:** ^1^ Department of Psychiatry and Psychotherapy, Medical Centre – University of Freiburg, Faculty of Medicine University of Freiburg Freiburg Germany; ^2^ Center for Basics in NeuroModulation (NeuroModulBasics), Faculty of Medicine University of Freiburg Freiburg Germany; ^3^ Division of Diabetes, Endocrinology and Gastroenterology, School of Medical Sciences University of Manchester Manchester UK; ^4^ Diabetes, Endocrinology and Metabolism Centre, Manchester Royal Infirmary Manchester University NHS Foundation Trust, Manchester Academic Health Science Centre Manchester UK; ^5^ Department of Clinical Neuroscience Karolinska Institutet Stockholm Sweden; ^6^ Department of Psychiatry Oxford University Oxford UK; ^7^ Sleep and Circadian Neuroscience Institute (SCNi), Nuffield Department of Clinical Neurosciences University of Oxford Oxford UK

**Keywords:** diffusion MRI, DTI, NODDI, sleep health, UK Biobank, white matter integrity

## Abstract

Many people experience impaired sleep health, yet knowledge about its neurobiological correlates is limited. As previous studies have found associations between white matter integrity and several sleep traits, white matter integrity could be causally implicated in poor sleep health. However, these studies were often limited by small sample sizes. In this study, we examine associations between multiple indices of white matter integrity and sleep health in 29,114 UK Biobank participants. Late chronotype, daytime sleepiness, insomnia symptoms and, most extensively, long sleep duration were independently associated with diffusion MRI markers of reduced white matter integrity. Previous findings showing an association between insomnia symptoms and decreased fractional anisotropy (FA) in the anterior internal capsule could not be replicated. To our knowledge, the current analysis is the first study to find an association between long sleep duration and impaired microstructural white matter integrity. Previous assumptions concerning the role of white matter integrity for insomnia are challenged.

## 1 Introduction

Poor sleep health is a common complaint (Dalmases et al. 2018), with insomnia symptoms alone affecting about 30% of adults worldwide (Morin et al. 2015). Moreover, poor sleep health has been linked to various adverse health outcomes (Hale et al. 2020). Cardiovascular diseases, cognitive dysfunction, metabolic abnormalities and increased mortality risk have all been associated with sleep traits including insomnia symptoms (Li et al. 2014; Troxel et al. 2018; Wardle‐Pinkston et al. 2019), short or long sleep duration (Cappuccio et al. 2011; Kyle et al. 2017; Liu et al. 2017; Lo et al. 2016), daytime sleepiness (Empana et al. 2009; Hays et al. 1996; Newman et al. 2000), and morning or evening chronotype (Kyle et al. 2017; Frost et al. 2009; Huang and Redline 2019). Particularly, insomnia disorder has also been associated with adverse mental health outcomes like depression, anxiety and substance abuse (Hertenstein et al. 2019). The mechanisms underlying these associations are not yet well understood, but results from previous research suggest that neurostructural abnormalities may play a crucial role. For example, reduced integrity of brain white matter has been associated with both impaired sleep (see below), cognitive decline, and psychiatric problems (Filley 2005). Understanding the relationships between white matter integrity and sleep traits may help to explain links between white matter integrity and health problems. Better knowledge of the neurobiological correlates of sleep traits could then lead to new treatments and preventive approaches.

### 1.1 White Matter Integrity and Sleep

White matter consists of billions of fibres which connect all brain regions, allowing them to function in neural networks. Intact white matter ensures quick neuronal conduction, which is essential for normal brain function (Filley 2005). Accordingly, loss of structural integrity of white matter leads to a disconnection of neural networks and therefore impaired information processing. Consequently, individuals with white matter lesions (e.g., multiple sclerosis) often experience cognitive dysfunction, emotional distress, or both (Filley 2005). Complementary to macroscopically visible white matter lesions, more subtle changes to white matter microstructure can be detected using diffusion magnetic resonance imaging (dMRI). By then applying a diffusion tensor (DTI) or the *neurite orientation dispersion and density imaging* (NODDI) model (Zhang et al. 2012) to the dMRI data, estimates of white matter integrity can be derived, such as fractional anisotropy (FA), axial diffusivity (AD), mean diffusivity (MD), radial diffusivity (RD), intra‐cellular volume fraction (ICVF), isotropic volume fraction (ISOVF), or oriental dispersion (OD; see Supplementary method section for more details on dMRI).

Changes in white matter integrity have consistently been associated with different sleep traits, for example, insomnia (Bresser et al. 2020; Grau‐Rivera et al. 2020; Kang et al. 2018; Li et al. 2016; Spiegelhalder et al. 2014), short sleep duration (Grumbach et al. 2020; Khalsa et al. 2017; Kocevska et al. 2019; Takeuchi et al. 2018; Yaffe et al. 2016), late chronotype (Rosenberg et al. 2014) and daytime sleepiness (Chondrogiorgi et al. 2016; Koller et al. 2020; Matsui et al. 2006). These changes usually involve reduced fractional anisotropy or altered radial, axial or mean diffusivity throughout the whole brain. In the case of insomnia, one specific brain region stands out, the anterior limb of the internal capsule—with previous research showing reduced fractional anisotropy or other altered dMRI indices (Bresser et al. 2020; Grau‐Rivera et al. 2020; Li et al. 2016; Spiegelhalder et al. 2014). While it is assumed that impaired sleep is associated with impaired white matter integrity, correlational studies that underpin this assumption show inconsistencies, making it challenging to draw clear conclusions. When exploring dMRI indices beyond fractional anisotropy, the putative impact on white matter integrity manifests itself in diverse ways, often conflicting with one another. For instance, mean, radial and axial diffusivity are frequently examined dMRI indices, but their results have shown both decreases (Takeuchi et al. 2018) and increases (Yaffe et al. 2016) in participants with specific sleep traits, depending on the study. Importantly, most of these studies have small sample sizes (around 50 or fewer participants).

### 1.2 Aims

The current study aims to conjointly investigate the associations between different dimensions of sleep health and white matter integrity. We limited ourselves to features of sleep that have previously been shown to be important for overall health, which is why we conceptualised sleep health as proposed by Buysse (2014). This model defines sleep health on a continuum from poor to good sleep health by combining the sleep health dimensions of sleep continuity, sleep quality, sleep duration, sleep timing and daytime sleepiness. Leaning on these sleep health dimensions, we made use of the sleep health variables insomnia symptoms, sleep duration, chronotype and daytime sleepiness as proxies available in the UK Biobank. Since each of these sleep health dimensions independently predicts poor health, it is advantageous to study their effects on the human brain in combination. Specifically, we aimed to (1) assess whether FA decreases in the anterior internal capsule in individuals with insomnia symptoms are replicable in a large population‐based sample, and (2) thoroughly investigate the relationship between sleep health variables and a broad set of DTI and NODDI parameters throughout a set of white matter tracts. We hypothesised that (A) insomnia symptoms are related to reduced FA values in the anterior internal capsule and that (B) there are independent associations between sleep health variables (insomnia symptoms, sleep duration, chronotype and daytime sleepiness) and different DTI and NODDI indices of white matter integrity. Unlike previous experimental studies, using the UK Biobank database enables the examination of multiple dimensions of sleep health and their relationship with white matter integrity with high statistical power.

## 2 Methods

### 2.1 Participants

The UK Biobank is a large prospective epidemiological study including over 500,000 adults between ages 40 and 69 years (Sudlow et al. 2015). From 2014 on, a subset of participants underwent multimodal MRI scanning. The current data set includes 37,441 participants with completed diffusion MRI scans (first imaging visit, *n* = 37,441 for DTI measures; *n* = 37,339 for NODDI measures; retrieved from data release June 2020). We included between 26,003 and 31,770 participants in different analyses after excluding participants with neurological disorders (*n* = 894; see Table S1 for details), sleep apnea (*n* = 155) and missing data in predictor variables (see Table S1).

The UK Biobank research procedures were approved by the NHS National Research Ethics Service (Ref. 11/NW/0382), and all participants gave written informed consent. This analysis was conducted under UK Biobank application number 6818 and has been officially preregistered at Open Science Framework (https://osf.io/dt43x). Preregistration was completed on the 21st of October in 2020; the UK Biobank data were subsequently downloaded on the 25th of October 2020. All analyses were based on a priori formulated hypotheses and conducted with R. Code will be made available on OSF upon publication.

### 2.2 Sleep‐Related Variables

To assess insomnia symptoms, participants were asked ‘Do you have trouble falling asleep at night or do you wake up in the middle of the night?’ with responses ‘never/rarely’, ‘sometimes’ and ‘usually’. Participants were categorised as having insomnia symptoms if they answered ‘usually’ to this question, with the remaining participants categorised as not having insomnia symptoms. Sleep duration was assessed by asking participants ‘About how many hours sleep do you get in every 24 hours? (please include naps)’ and recorded in full hours. In light of previously established U‐shape relationships with health and cognition (Lo et al. 2016), sleep duration was categorised as short (< 7 h), normal (7–9 h) or long (> 9 h) based on guidelines (Watson et al. 2015a). Chronotype was assessed with the question ‘Do you consider yourself to be…’: ‘definitely a “morning” person’, ‘more a “morning” than “evening” person’, ‘more an “evening” than “morning” person’, ‘definitely an “evening” person’. The two middle responses were collapsed into an ‘intermediate’ chronotype category. Excessive daytime sleepiness was assessed using the following question, ‘How likely are you to doze off or fall asleep during the daytime when you don't mean to? (e.g., when working, reading or driving)’ with responses ‘never/rarely’, ‘sometimes’ and ‘often’. Participants were categorised as having excessive daytime sleepiness if they answered ‘often’ to this question, while the remaining participants were classified as the group without excessive daytime sleepiness. Of note, the definition of sleep‐related variables was in line with recent studies on sleep health, neurocognitive function and amygdala reactivity from our group (Kyle et al. 2017; Schiel et al. 2022).

### 2.3 Demographic Data

Age, sex, level of education, and neighbourhood‐level socioeconomic status (measured by the Townsend index of material deprivation, TDI) were used as demographic covariates in the analyses. Educational qualifications were recorded and dichotomised according to whether or not participants held a college/university degree. Socioeconomic status was log‐transformed due to skewed distribution using a ln(x + 7) equation (minimum of non‐transformed index: −6.26).

### 2.4 Other Clinical Data

Current medications were recorded during an interview by a research nurse, and included sleep medication (sedatives and hypnotics, see Table S2), other psychotropic medication (mood stabilisers, antidepressants, antipsychotics, see Table S3) and/or antihypertensive medication (ACE inhibitors, angiotensin II blockers, beta blockers, calcium channel blockers, diuretics, see Table S4). Current depressive symptoms were assessed using the following question, ‘Over the past two weeks, how often have you felt down, depressed or hopeless?’ with the following response options: ‘not at all’, ‘several days’, ‘more than half the days’ or ‘nearly every day’. For the purpose of the present analyses, those scoring ‘several days’, ‘more than half the days’ or ‘nearly every day’ were coded in the ‘depressive symptoms’ category, while those scoring ‘not at all’ were coded in the ‘no depressive symptoms’ category. Body mass index (BMI) served as further covariate in the analyses.

The presence of hypertension or other cardiovascular diseases was classified by the research nurse using the UK Biobank ‘non‐cancer illness’ and ‘vascular/heart problems diagnosed by doctor’ codes (see Table S5 for details). In the latter, participants were asked if a doctor had ever told them they had any of the following conditions: heart attack, angina pectoris, stroke or high blood pressure. Of note, the ‘non‐cancer illness’ code was also used (alongside the ‘cancer’ code) to determine whether participants presented sleep apnea or neurological diseases (see Table S6 for details) for exclusion purposes.

### 2.5 Magnetic Resonance Imaging (MRI)

Diffusion MRI acquisition and pre‐processing were performed by the UK Biobank team; full details of the applied protocols have been described elsewhere (Alfaro‐Almagro et al. 2018). This analysis used the imaging‐derived phenotypes (IDPs) produced by the UK Biobank team.

Images were collected on identical Siemens Skyra 3.0 T scanners using a Stejskal–Tanner pulse sequence. For each of the two diffusion‐weighted shells (*b* = 1000 and 2000 s/mm^2^), 50 distinct diffusion‐encoding directions were acquired. After correcting for eddy currents and head motion, the *b* = 1000 s/mm^2^ shell was fed into the DTI fitting tool to extract diffusion tensor model parameters (FA, MD, RD, AD). In addition, the whole two‐shell dMRI data was fed into NODDI modelling, which estimated microstructural properties of tissue (ICVF, ISOVF, OD). Both models were fitted voxel‐wise, producing maps of DTI and NODDI outputs. Cross‐subject alignment of white matter paths was achieved through tract‐based spatial statistics, which project subjects' local peak FA values onto a standard‐space FA skeleton. The resulting standard‐space warp was then also applied to all other DTI/NODDI output maps. By means of the JHU ICBM DTI‐81 white matter label atlas (Wakana et al. 2004), 48 white matter tracts were identified and their average DTI/NODDI parameters extracted as an IDP for each subject. In addition, the participants' brain volume (volumes of grey and white matter obtained by T1 structural MRI brain images to account for potential differences in white matter structure depending on brain size Wang et al. 2008) was included in the analysis as a covariate.

### 2.6 Statistical Analysis

For descriptive data presentation, mean (SD) values were used as well as the absolute numbers (%) of participants reporting specific sleep or medical characteristics (see Table 1). Response options ‘do not know’ or ‘prefer not to answer’, were classified as missing values. The association between insomnia symptoms and FA in the anterior limb of the internal capsule (hypothesis A) was analysed using two linear models (left and right internal capsule) with insomnia symptoms as predictor and FA values of the left and right anterior internal capsule as dependent variable. Covariates included in the three linear models tested were: model 1, age, sex, BMI, and brain volume; model 2, model 1 covariates plus level of education, TDI, sleep medication, depressive symptoms, and psychopharmacological treatment; model 3, model 2 covariates plus hypertension, antihypertensive medication, and cardiovascular disease. Accounting for the testing of two hypotheses (A and B), the Bonferroni‐adjusted alpha level was *α* = 0.025. As hypothesis A tested two brain regions, it was further adjusted to *α* = 0.0125 (0.025/2; one‐tailed) within this hypothesis.

**TABLE 1:** Clinical characteristics (model 3, *n* = 29,115).

Sex	
Female	15,797 (54%)
Male	13,318 (46%)
Age (years)	63.2 ± 7.5
Insomnia symptoms	9109 (31%)
Sleep duration (hours)	7.2 ± 1.0
Short (< 7 h)	6880 (24%)
Medium (7–9 h)	21,907 (75%)
Long (> 9 h)	328 (1%)
Chronotype	
Morning	8072 (28%)
Intermediate	18,225 (63%)
Evening	2788 (10%)
Daytime Sleepiness	6486 (22%)
BMI (kg/m^2^)	26.4 ± 4.4
Education, college degree	15,231 (52%)
Townsend index of material deprivation	1.5 ± 0.5
Sleep medication	67 (0.2%)
Depressive symptoms	5148 (18%)
Psychotropic medication	1759 (6%)
Hypertension	5557 (19%)
Antihypertensive medication	6300 (22%)
Cardiovascular disease	2242 (8%)
CSH (exploratory analysis)	4.1 ± 0.9

*Note*: Data are *N* (%) or mean ± SD.

Abbreviations: BMI = body mass index, CSH = composite measure of sleep health.

The association between sleep health variables (insomnia symptoms, short/long sleep duration, early/late chronotype and daytime sleepiness) and dMRI parameters (FA, MD, AD, RD, ICVF, OD, ISOVF) in all 48 white matter regions (hypothesis B) was analysed using multiple linear models. For sleep duration and chronotype, the intermediate categories (normal sleep duration/intermediate chronotype) served as reference categories.

All four sleep health variables served as predictor variables (six predictor variables in total due to the two levels of long/short sleep duration and early/late chronotype) and were combined with each IDP (e.g., MD of fornix) as dependent variables, resulting in a total of 334 linear models per predictor variable (46 brain regions x 7 dMRI parameters +2 anterior limbs of internal capsule x 6 dMRI parameters excluding FA). Accordingly, the Bonferroni‐adjusted alpha level was *α* = 7.5 × 10^−5^ (0.025/334; two‐tailed). In line with the analysis of the anterior internal capsule (above), covariates varied with the three models tested.

#### 2.6.1 Exploratory Analysis

In addition to our pre‐registered analyses, we tested whether a composite measure of sleep health was associated with white matter integrity. For this, we replaced all sleep‐related variables of model 3 (insomnia symptoms, sleep duration, chronotype, daytime sleepiness and sleep medication) with one composite measure of sleep health (CSH). As proposed by previous research (Dong et al. 2019), participants' CSH score was derived from the abovementioned sleep‐related variables (see Figure S1 for more details) and in line with our previous research (Schiel et al. 2022). Note that, to make the results more easily comparable to those of model 1–3, we changed the direction of the CSH: in contrast to previous literature (Schiel et al. 2022; Dong et al. 2019), where a high CSH indicates good sleep health, we have reversed the interpretation so that a high CSH now reflects poor sleep health. The Bonferroni‐adjusted alpha level was set to 0.05/336.

#### 2.6.2 Sensitivity Analyses

We explored the implications of our relatively strict Bonferroni correction by comparing the original results with the results of a more liberal FDR correction. Moreover, we conducted a further sensitivity analysis in which all patients with an in‐patient ICD‐10 diagnosis of any neurological disease (G00.0–G99.8) or any psychiatric disorder (F00.0–F99.0) were excluded. In addition to the covariates of model 3, the scanner site was also included as an additional covariate in this analysis; the Bonferroni‐adjusted alpha level was set to 0.05/336.

## 3 Results

Clinical characteristics are illustrated in Table 1. The cohort was comparable with the general population in terms of sex and education (46% male, 52% college degree), though considerably older, with a mean age of 63.2 years. The average sleep duration of 7.2 h lies within the scope of previously reported subjective sleep duration (Lauderdale et al. 2008). Insomnia symptoms were reported by 31%, yet only 0.2% of the sample reported taking any sleep medication.

Hypothesis A *Insomnia symptoms and FA in the anterior internal capsule*.

After adjusting for multiple comparisons, all three models yielded no statistically significant relationships between insomnia symptoms and FA in the anterior internal capsule, neither on the left nor on the right side (Table 2).

Hypothesis B *Sleep health and white matter integrity*.

**TABLE 2:** Relationships between insomnia symptoms and fractional anisotropy in the left and right anterior internal capsule (AIC), uncorrected *p*‐values (alpha level at 0.0125).

	Left AIC		Right AIC	
	*β*‐coefficient	*p*	*β*‐coefficient	*p*
Model 1	8.6 × 10^−5^	0.62	−2.0 × 10^−4^	0.24
Model 2	2.5 × 10^−5^	0.53	−1.5 × 10^−4^	0.31
Model 3	1.3 × 10^−4^	0.67	−4.1 × 10^−5^	0.45

**FIGURE 1:**
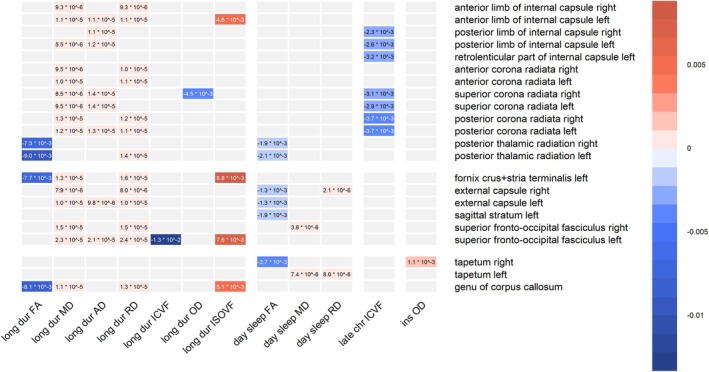
Significant beta weights by sleep health dimension, dMRI parameter and brain region (model 3). The magnitude of the significant beta weights is illustrated by colour, the exact value displayed. The x‐axis displays significant sleep health variables (long dur = long sleep duration; day sleep = daytime sleepiness; late chr = late chronotype; ins = insomnia symptoms) along with the affected dMRI parameters (AD = axial diffusivity; FA = fractional anisotropy; ICVF = intra‐cellular volume fraction; ISOVF = isotropic volume fraction; MD = mean diffusivity; OD = oriental dispersion; RD = radial diffusivity), brain regions are listed on the y‐axis and sorted into four blocks containing brain stem, projection, association and commissural tracts from top to bottom.

**FIGURE 2:**
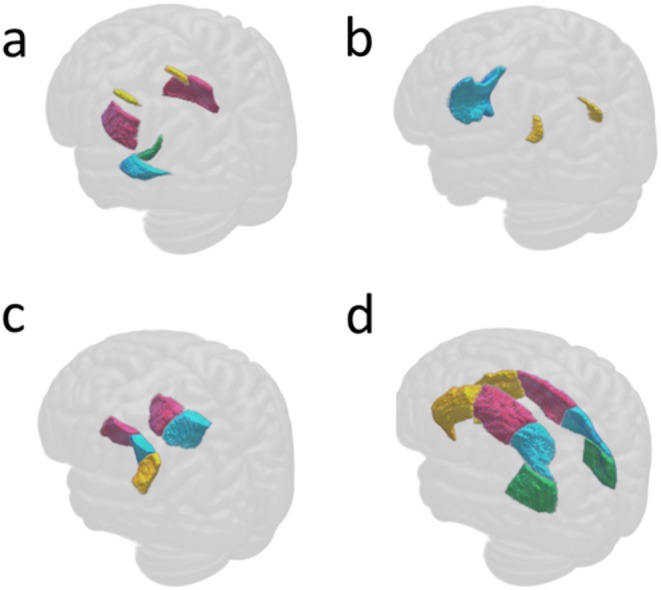
Brain plot. White matter tracts that showed a significant association to any sleep health variable in model 3 (a) association fibres: External capsule (red), fornix crus + stria terminalis left (green), sagittal stratum left (blue) and superior fronto‐occipital fasciculus (yellow); (b) commissural fibres: Genu of corpus callosum (blue) and tapetum (yellow); (c) internal capsule (projection fibres): Anterior (red), posterior (blue) and left retrolenticular internal capsule (yellow) (d) projection fibres: Anterior (yellow), superior (red) and posterior (blue) corona radiata, posterior thalamic radiation (green).

Significant beta weights of all three models are presented numerically in the corresponding cells in Figures [Link], [Link]: results that were significant after FDR correction but not after Bonferroni correction are marked as ‘FDR’. Significant beta weights of model 3 are also shown in the corresponding cells in the simplified Figure 1. Additionally, Figure 2 presents the anatomy of white matter tracts that were significantly associated with sleep health variables. Out of all six predictor variables that were studied, only four showed significant associations with white matter integrity. As shown in Figure 2, the most extensive associations were found between white matter integrity and long sleep duration, followed by associations with daytime sleepiness, late chronotype, and finally only one significant white matter alteration in those with insomnia symptoms. Across several white matter tracts, long sleep duration and daytime sleepiness showed a decrease in FA, accompanied by an increase in MD, AD and/or RD. In contrast, late chronotype was exclusively associated with decreased ICVF and insomnia was solely associated with increased OD within the right tapetum. In total, 24 out of the 48 white matter tracts showed significant alterations of their microstructure in relation to at least one sleep health variable. There were no significant findings in the 10 brainstem white matter tracts (first block on the y‐axis in Figures [Link], [Link]), with significant changes present only in the projection, association, and commissural tracts (corresponding to the three lower blocks on the y‐axis in Figure 2). The vast majority of the significantly altered white matter tracts (22 out of 24) showed this relationship to sleep health consistently across all three models (right sagittal stratum and left superior longitudinal fasciculus were rendered insignificant in model 3 and are therefore not shown in Figure 1). The same consistency was found for the affected sleep health variables: in all three models, short sleep duration and early chronotype showed no significant association with white matter changes, and insomnia symptoms showed only one significant result in model 3 (as discussed above). Affected brain tracts, sleep health variables, and DTI/NODDI parameters thus showed a relatively consistent pattern across all three models, namely FA and ICVF decreases along with AD/RD/MD/ISOVF increases in projection, association, and commissural tracts in those with long sleep duration, late chronotype, and daytime sleepiness.

### 3.1 Exploratory Analysis: Composite Sleep Health

**FIGURE 3:**
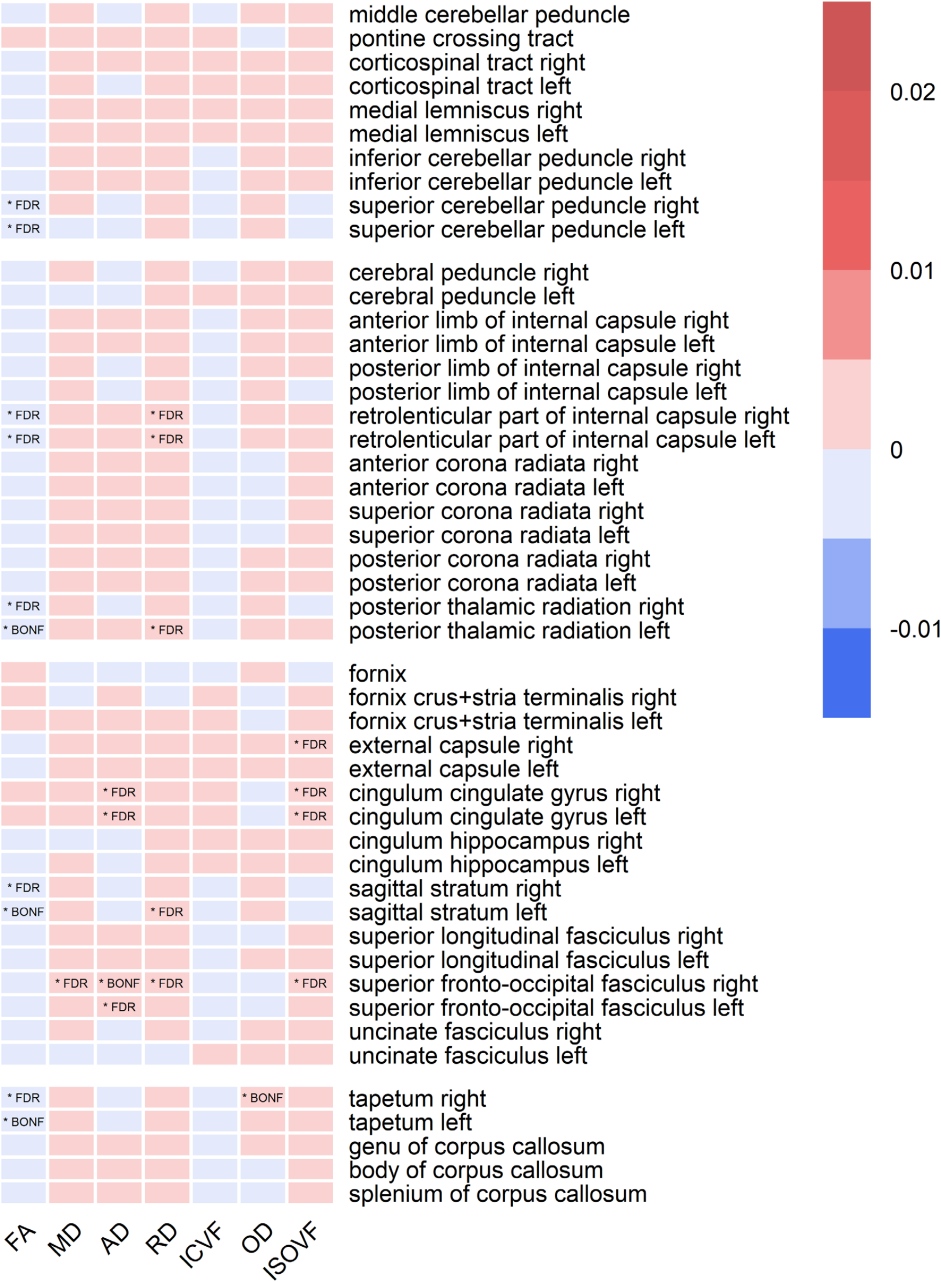
Significant associations of composite sleep health (exploratory analysis). Results that were significant after Bonferroni correction are marked as ‘*BONF’ in the corresponding cell, results that were significant after FDR correction but not after Bonferroni correction are marked as ‘*FDR’. The x‐axis displays the dMRI parameters (AD = axial diffusivity; FA = fractional anisotropy; ICVF = intra‐cellular volume fraction; ISOVF = isotropic volume fraction; MD = mean diffusivity; OD = oriental dispersion; RD = radial diffusivity), brain regions are listed on the y‐axis and sorted into four blocks containing brain stem, projection, association and commissural tracts from top to bottom.

Figure 3 presents all significant associations for the analysis of the CSH. Results that were significant after Bonferroni correction are marked as ‘* BONF’ in the corresponding cell; results that were significant after FDR correction but not after Bonferroni correction are marked as ‘* FDR’. Figure S5 presents all significant beta weights numerically in the corresponding cells. Results that were significant after FDR correction but not after Bonferroni correction are marked as ‘FDR’. Although the general trend of white matter changes is similar to that of models 1–3 (in terms of white matter tracts affected and direction of white matter changes), the exploratory analysis yielded few significant results, and most of these were only significant after the less stringent FDR correction. Thus, compared to models 1–3, the number and strength of significant results were less pronounced than for long sleep duration, daytime sleepiness, and late chronotype, but more pronounced than for short sleep duration, early chronotype, and insomnia.

### 3.2 Sensitivity Analyses

As can be seen in Table S7, applying a more liberal FDR correction resulted in more significant white matter tracts. This increase in significant results generally emphasised the pattern already apparent from the set of results that were significant after Bonferroni correction: sleep health variables that previously had not shown any significant relationship with white matter changes did not show a significant relationship after FDR correction either (with three exceptions for short sleep duration). Vice versa, white matter tracts and sleep health variables that had previously shown a significant relationship after Bonferroni correction were only more so after applying FDR correction. This can best be captured visually by inspecting Figures [Link], [Link], where cells labelled “FDR” and cells with beta weights (indicating significance after Bonferroni adjustment) tend to cluster within the same sleep health variable and/or white matter tract. Notably, though, and in contrast to models 1–3, with the FDR correction some tracts of the brain stem showed significant associations with sleep health variables, mainly concerning the superior, middle, and inferior cerebellar peduncles.

**FIGURE 4:**
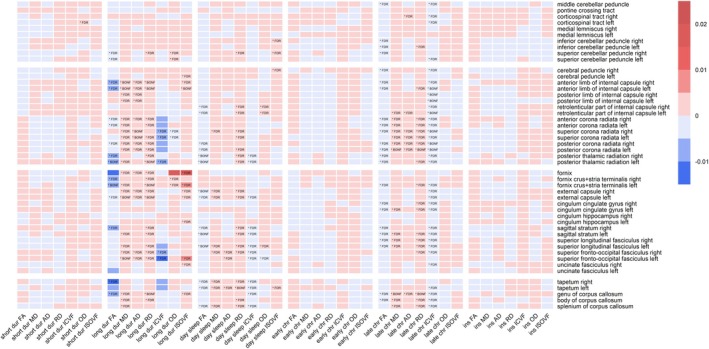
Significant associations by sleep health dimension, dMRI parameter and brain region (sensitivity analysis). Results that were significant after Bonferroni correction are marked as ‘*BONF’ in the corresponding cell, results that were significant after FDR correction but not after Bonferroni correction are marked as ‘*FDR’. The x‐axis displays the sleep health variables (short dur = short sleep duration; long dur = long sleep duration; day sleep = daytime sleepiness; early chr = early chronotype; late chr = late chronotype; ins = insomnia symptoms) along with the dMRI parameters (AD = axial diffusivity; FA = fractional anisotropy; ICVF = intra‐cellular volume fraction; ISOVF = isotropic volume fraction; MD = mean diffusivity; OD = oriental dispersion; RD = radial diffusivity), brain regions are listed on the y‐axis and sorted into four blocks containing brain stem, projection, association and commissural tracts from top to bottom.

Figure 4 presents all significant associations for the second sensitivity analysis, that is, exclusion of psychiatric/neurological disorders and inclusion of scanner site as an additional covariate. Results that were significant after Bonferroni correction are marked as ‘* BONF’ in the corresponding cell; results that were significant after FDR correction but not after Bonferroni correction are marked as ‘* FDR’. Figure S6 presents all significant beta weights numerically in the corresponding cells. Results that were significant after FDR correction but not after Bonferroni correction are marked as “FDR”. Compared to model 3, the sensitivity analysis resulted in a reduction of significant results for long sleep duration and daytime sleepiness, but an increase for late chronotype (see also Table S7). It is worth noting that the reduction in significant results for long sleep duration and daytime sleepiness was mainly due to the exclusion of neurological and psychiatric conditions, and not due to the inclusion of scanner site. Late chronotype, however, gained significant results both when including scanner site and when excluding neurological and psychiatric conditions. In general, the pattern of white matter changes was similar to that observed in models 1–3, including changes in brainstem tracts when FDR correction was included as described above. This tendency was particularly pronounced in those with late chronotype. In conclusion, neurological and psychiatric conditions appear to mediate some of the correlations between long sleep duration, daytime sleepiness, and white matter changes, while presumably suppressing them in late chronotype.

### 3.3 Comparison of Models

**FIGURE 5:**
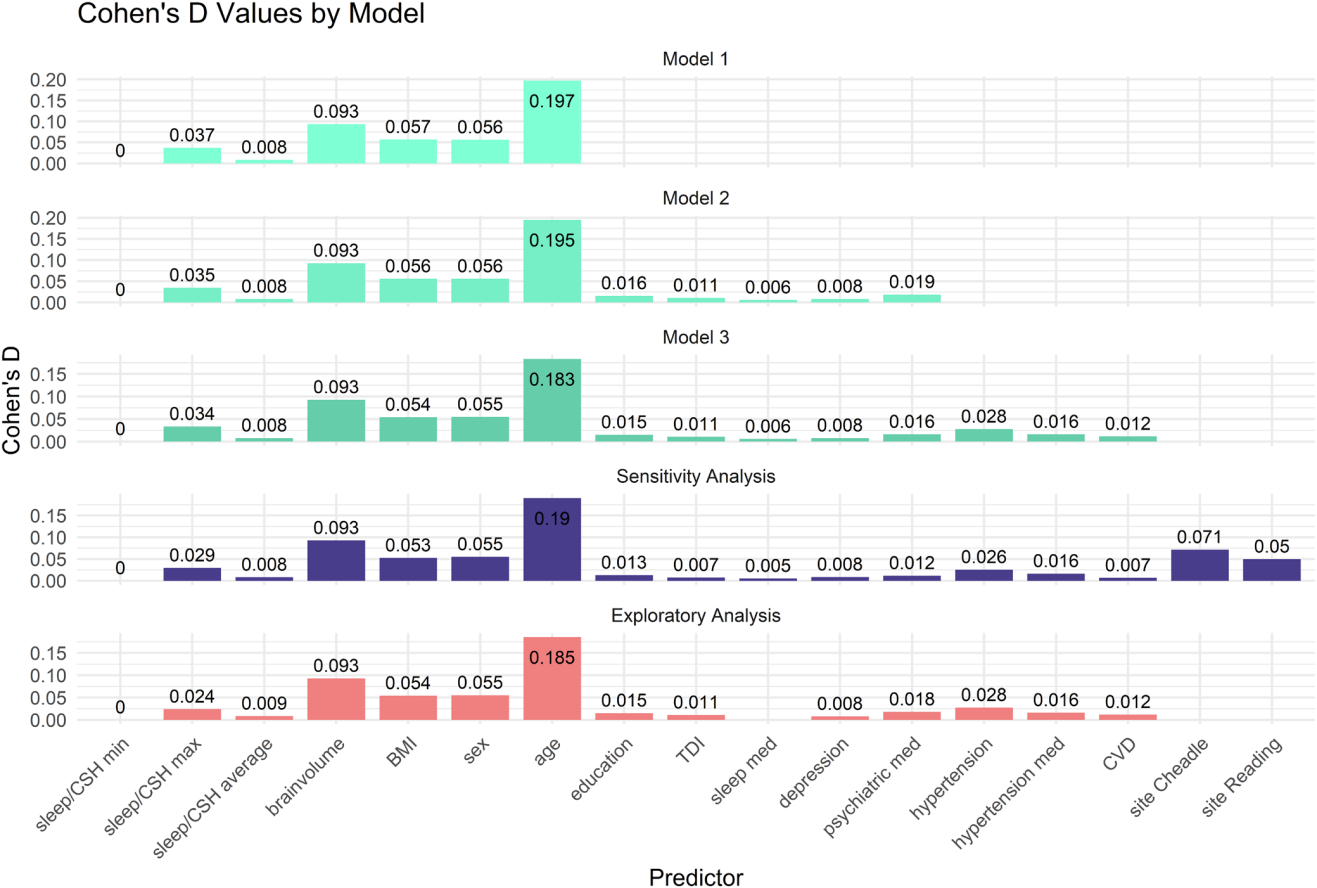
Model comparison of average Cohen's *D* values. Displayed are minimum and maximum absolute Cohen's *D* values as well as the average Cohen's *D* value across all dependent variables and all sleep health variables (respectively the CSH‐score within the exploratory analysis). Furthermore, average absolute Cohen's *D* values of the other predictors of all five models are tested.

The average explained variance of the three hypothesis‐driven models, the model for the exploratory analysis, and the model for the second sensitivity analysis are presented in Table 3; Cohen's *D* of these models are presented in Figure 5. The average rounded absolute Cohen's *D* for the sleep health variables was almost identical for all five models (Cohen's *D* = 0.008–0.009). Thus, the overall effect size of all sleep health variables was comparable to the effects of depression (Cohen's *D* = 0.008) or cardiovascular disease (Cohen's *D* = 0.007–0.012), but smaller than the effects of, for example, education (Cohen's *D* = 0.013–0.016) or hypertension (Cohen's *D* = 0.026–0.028).

**TABLE 3:** Comparison of the predictive value of all five models. Displayed are the sum of *R* ^2^ of all predictors across all dependent variables used in the corresponding models, the average *R* ^2^, the total variance of the dependent variables, the sum of *R* ^2^ divided by the total variance of the dependent variables, and the *F*‐ and *p*‐values for the comparison of average *R* ^2^ between model 2 and model 1, between model 3 and model 2, between the sensitivity analysis and model 3, and between model 3 and the exploratory analysis (alpha level at 0.05).

Model	*R* ^2^ sum	*R* ^2^ average	Total variance	*R* ^2^ sum/total variance	*F*‐value	*p*
Model 1	27.873	0.083	0.24	115.982		
Model 2	27.994	0.083	0.24	116.489	0.026	1.000
Model 3	29.188	0.087	0.24	121.457	0.415	0.742
Sensitivity	33.671	0.1	0.238	141.636	4.73	0.030 *
Exploratory	29.003	0.086	0.24	120.687	0.048	0.996

* Indicates significance with an alpha level set at 0.05.

In terms of the average *R* ^2^, model 2 did not show a significantly better model fit than model 1, and model 3 did not show a significantly better model fit than model 2 (see Table 3). However, the sensitivity analysis showed a significantly better fit to the data when compared to model 3. This better fit is likely due to the inclusion of the scanner site in the sensitivity analysis, which showed a substantial effect size (Cohen's *D* = 0.071 and 0.050, see Figure 5) and rendered the *F*‐test significant even without excluding participants with neuropsychiatric conditions.

In the exploratory analysis, the calculated CSH score showed only a marginally higher Cohen's *D* (*D* = 0.009) than the average sleep health variables from models 1–3 (*D* = 0.008). Considering that the sleep medication variable (*D* = 0.006) was included in the CSH score, this increase in Cohen's *D* from 0.008 to 0.009 is not very striking. Thus, the CSH score does not seem to provide any additional predictive value compared to a simple average of the contributing predictors.

## 4 Discussion

This study set out to investigate the relationship between different aspects of sleep health and white matter integrity. The first hypothesis that FA is reduced in the anterior internal capsule of individuals with insomnia symptoms was not supported. However, in line with our second hypothesis, several independent correlations between sleep health dimensions and indices of white matter integrity were observed. Arguably, the effect sizes of the significant results were relatively small, ranging from Cohen's *D* 0.023 to 0.034 (model 3). However, due to the imprecise measurement of the many predictors and outcomes (e.g., sleep duration was only recorded in full hours), the true effect sizes may be considerably underestimated.

### 4.1 Affected White Matter Tracts

White matter tracts that were significantly associated with sleep health variables were not restricted to any particular brain hemisphere or brain region. Rather, sleep health variables correlated with wide‐spanning white matter tracts. These encompass large projection, association, and commissural fibres, leaving out mainly tracts of the brainstem. Most correlations were found in projection fibres, which connect cortical areas with basal parts of the brain. Among these projection fibres are the corona radiata, posterior thalamic radiation, and all parts of the internal capsule. Commissural fibres (like the corpus callosum and tapetum) and association fibres (like the external capsule, sagittal stratum, fornix + stria terminalis and superior fronto‐occipital fasciculus) only showed associations to select sleep health variables and, if so, in many cases one‐sided. Tracts of the brainstem and cerebellum did not show any association with sleep health variables after Bonferroni correction. After the less stringent FDR correction, mainly tracts in the cerebellar peduncles were rendered significant.

This observation is consistent with the retrogenesis hypothesis of white matter that ontogenetically early myelinated white matter tracts such as the brain stem and cerebellum (Barkovich et al. 1988) are less susceptible to deterioration than white matter tracts that are myelinated later in life. This principle can be summarised in the ‘last‐in‐first‐out’ principle (Slater et al. 2019). The underlying reason may be that later‐developing pathways are more thinly myelinated and the comparatively high metabolic activity of their oligodendrocytes makes them more susceptible to aggregating damaging effects (Cox et al. 2016; Glasser et al. 2014). Because they develop relatively late in life, anterior and association pathways are more heavily affected (Slater et al. 2019; Cox et al. 2016; Salat et al. 2005). Projection tracts are thought to myelinate relatively early in life (Cox et al. 2016), and therefore should have displayed relatively stable white matter. However, the coronae radiatae and internal capsules are primarily composed of projection tracts, and yet, contrary to theory, they showed consistent changes in our sample. This inconsistency with the ‘first in last out’ principle is not novel. It has been observed previously in the internal capsule of insomnia patients (Grau‐Rivera et al. 2020; Li et al. 2016; Spiegelhalder et al. 2014; Sexton et al. 2017) (which led us to formulate hypothesis A) and in the ageing brain (Salat et al. 2005). Despite this exception to the rule, it would be possible that, in general, poor sleep puts a strain on white matter integrity comparable to ageing, and therefore has a similar pattern of decline. In this vein, other markers of brain ageing have been described in association with poor sleep: White matter hyperintensities, small lesions in the white matter that accumulate over a lifetime (d' Arbeloff et al. 2019), have been associated with late chronotype, daytime sleepiness, and both short and long sleep duration (Ning et al. 2023). Another study found lower white and grey matter volumes and even an increased brain age gap itself in long sleepers (Stolicyn et al. 2024). Of course, these associations are not always clear‐cut and may interact with age itself (Schiel et al. 2023). Consistent with the findings of this study, however, the three other recent UK Biobank studies on sleep and white matter also reported a focus on thalamic (i.e., projection) and association tracts, as well as the somewhat surprising associations with long sleep duration (Ning et al. 2023; Stolicyn et al. 2024; Wang et al. 2024).

### 4.2 Affected dMRI Parameters

As can be seen in the colour‐coding of Figures 1 and [Link], [Link], across all sleep health dimensions and white matter tracts, significant dMRI parameter changes indicated the same microstructural alterations, namely a decrease in FA and ICVF and an increase in MD, AD, RD and ISOVF. As an exception to this pattern, OD was both increased with insomnia symptoms and decreased with long sleep duration. Moreover, in the exploratory analysis, worse sleep health was connected with lower FA and higher AD.

Indices derived from DTI or NODDI models are not always straightforward to interpret as they can be influenced by different biological processes. A tissue's degree of anisotropy can be influenced by axon density, its alignment, and axonal diameter, as well as axonal membrane permeability and myelin structure (Beaulieu 2002; Tournier et al. 2011). Just as FA tends to increase in maturing brains, MD is usually lower in fully myelinated brains (Engelbrecht et al. 2002). As high MD and low FA both show a connection to neurodegenerative processes (Horsfield and Jones 2002), these alterations are often broadly interpreted as a loss of white matter integrity, though the exact mechanistic origin remains unclear. Both DTI alterations were present in our sample, suggesting impaired white matter. Concerning the NODDI indices, increased ISOVF along with decreased ICVF was found. This indicates elevated volume fraction with isotropic diffusion and compatibly diminished intracellular volumes. Since OD measures mirroring the degree of axon misalignment pointed to opposite directions (also when including non‐significant tracts), axon dispersion remains elusive. This indicates that the white matter alterations are not mainly driven by (mis)alignment of neurons, but rather by the loss of axon diameter or density as suggested by the ISOVF and ICVF measures. All these do not exclude a change in the membrane permeability or myelin structure as myelin has been shown to be highly responsive to the neuron it ensheaths (Vivo and Bellesi 2019). Taken together, the results of the current study suggest that sleep health covaries with a global deterioration of cerebral white matter tracts, and this effect seems most pronounced in projection fibres.

### 4.3 Composite Sleep Health (CSH) and Subtyping

The CSH score revealed only a few alterations of white matter integrity (see Figure 3). They were consistent with the trends observed for individual sleep health dimensions, also when including results that were significant only with FDR correction. However, the CSH had no additional utility in predicting white matter alterations in poor sleepers. When reconsidering the conceptualisation of the CSH (Figure S1) in light of the results of models 1–3, the reasons for this become apparent: Compared to daytime sleepiness, late chronotype and long sleep duration, the sleep health dimensions early chronotype, short sleep duration, and insomnia play no significant role with respect to white matter integrity. However, they were equally considered in the calculation of the CSH. Likewise, sleep medication showed no significant association with dMRI data in our sample (model 3). The original CSH score on which we based our CSH score was associated with several health measures in adolescents (Dong et al. 2019). However, it was slightly different from our version (e.g., assessing six dimensions, partially unavailable in the UK Biobank). It may be that our version was suboptimal, as it also previously showed no correlation to amygdala reactivity (Schiel et al. 2022) and could potentially benefit from a specific adaptation to dMRI or fMRI data. When the appropriate aspects are included in such a CSH score, it could then shed light on the interactions between sleep health dimensions and demonstrate potential additive effects on brain structures. However, the general idea of a sleep health score is that it also reflects a person's overall state of health, which naturally should include a healthy brain structure. Adapting the CSH specifically to white matter would then fail to meet the original intent of a comprehensive approach.

In fact, our data suggested that each dimension of sleep health differed substantially in the degree of association with white matter integrity. Furthermore, excluding neuropsychiatric conditions in the sensitivity analysis reduced the significant findings for long sleep duration and daytime sleepiness but increased them for late chronotype. This illustrates that the presence of neuropsychiatric conditions may interact quite differently with different sleep health dimensions and associated white matter changes. Thus, aggregation across sleep health variables (such as with the CSH) and possibly different comorbid neuropsychiatric conditions could dilute and/or bias correlations with white matter integrity. For example, late chronotypes with a neuropsychiatric condition might differ substantially in white matter integrity from late chronotypes without these conditions, but when both groups are examined together, the effects (partially) cancel each other out. This line of thinking is not new, having been developed mostly in the context of insomnia and extending beyond white matter (Bresser et al. 2025) to include biomarkers such as EEG markers (Blanken et al. 2019) or functional connectivity (Ran et al. 2024). The classification of potential subtypes of insomnia has now moved away from a phenotypic categorisation based on sleep‐related features (e.g., sleep‐onset insomnia) in favour of underlying psychological mechanisms such as reward sensitivity and environment reactivity (Blanken et al. 2019). These subtypes, in turn, appear to be related to psychiatric conditions such as depression and anxiety (Blanken et al. 2019). Therefore, rather than aggregating across multiple sleep traits, it may be more fruitful to even distinguish between potential subtypes of individual sleep health dimensions. In the absence of a commonly accepted subcategorization to date, psychiatric conditions, particularly depression and anxiety, may be a good place to start.

### 4.4 Sleep Duration

The analysis of sleep duration showed broad white matter alterations with long sleep duration, yet no significant results with short sleep duration in any of the tested models. This comes as a surprise since previous research usually found an inverse relationship, where white matter integrity was reduced in short sleepers and preserved or improved in long sleepers (Grumbach et al. 2020; Khalsa et al. 2017; Kocevska et al. 2019; Yaffe et al. 2016). Unlike long sleep, short sleep has also been studied in experimental settings and therefore physiological models exist that could explain the link between short sleep and white matter alteration (Vivo and Bellesi 2019; Voldsbekk et al. 2020). However, when looking beyond the realm of dMRI research, not only short sleep but also long sleep has indeed been associated with adverse outcomes, in many cases even stronger than short sleep (Patel et al. 2006). Long sleep is associated with increased morbidity (e.g., diabetes, heart disease and stroke Patel et al. 2006), impaired cognition (Kyle et al. 2017; Lo et al. 2016) and overall increased mortality (Liu et al. 2017). As these epiphenomena of long sleep have been shown to be associated with impaired white matter integrity (Debette et al. 2010), it is not implausible that there may also be a connection between long sleep and reduced white matter integrity, especially since other recent studies have also shown this association (Ning et al. 2023; Stolicyn et al. 2024; Wang et al. 2024). Certainly, a clear physiological model that could explain this link is lacking so far.

Just like with the U‐shaped distribution of its comorbidities, sleep duration might be accompanied by decreased white matter integrity at both its extremes (as suggested by Ning et al. (2023)). This nonlinear connection might be overlooked when focusing on a linear analysis or could be lost depending on the categorisation into short and long sleepers. Clearly, our subgroup of long sleepers (more than 9 h, *n* = 328) comprised more extreme sleep durations than did our subgroup of short sleepers (6 h or less, *n* = 6880; see Table 1). This might have rendered short sleep duration nonsignificant in our sample. It should be noted, though, that the definitions of short or long sleep vary between different studies, and therefore results are difficult to compare and interpret.

### 4.5 Causality and Sensitivity Analysis

An important aspect that cannot be answered by this cross‐sectional study is causality. A common interpretation in this field of research is that impaired sleep health leads to white matter changes (Yaffe et al. 2016). In vitro research in rodents suggests that sleep fosters myelination while also behavioural stress (which may be associated with sleep complaints) can induce complex responses in oligodendrocytes (Vivo and Bellesi 2019). Likewise, experimental sleep deprivation in humans has been shown to affect dMRI indices (Elvsåshagen et al. 2015). Concerning long sleep, experimental data are scarce, and unlike short sleep, plausible biological models of how it might adversely affect human physiology are lacking so far (Watson et al. 2015b). Hypotheses include that a longer time spent with reduced body temperature and high levels of serum melatonin or cortisol could influence biological processes (Patel et al. 2006), such as white matter modelling. However, there is also evidence for reverse causality, as sleep disorders have been observed with pre‐existing white matter damage like multiple sclerosis or vascular dementia (Vivo and Bellesi 2019). Of course, other options exist as well: A high level of inflammation has been proposed as a common base of both short and long sleep and their associated increased mortality (Patel 2009); the brain's white matter could simply mirror this general inflammation (Favrais et al. 2011). Another possibility for a common third factor or mediator in both directions would be neurological and/or psychiatric disorders. However, to our knowledge, no previous study has examined the causal relationship between sleep health, psychiatric health, and white matter integrity. Only in the area of cognition did two studies suggest that sleep may affect cognition via white matter integrity (Grumbach et al. 2020; Rocklage et al. 2009). Using a similar approach as in these studies, mediation/moderation analysis or stepwise models could help to shed light on the causal relationship between sleep, white matter integrity, and neurological/psychiatric disorders before implementing elaborate longitudinal or experimental designs. Out of the few studies that tried to assess the direction of causality, two reject the notion that poor sleep quality leads to the observed white matter changes (Kocevska et al. 2019; Sexton et al. 2017). This slight tendency towards a reversed causality could be underlined by the fact that daytime sleepiness was the sleep health dimension impacted most (along with long sleep duration). Daytime dozing is unlikely to amount to a substantial prolongation of sleep duration and thereby reduce white matter integrity. Rather, it is conceivable that damaged white matter primarily leads to an increased need for sleep, expressing itself in either long sleep or daytime sleepiness. These considerations remain, of course, speculative but are nevertheless important. Concerning potential treatment or prevention, it is crucial to know whether sleeping habits present a risk factor for brain deterioration or whether sleep complaints are (partially) rooted in the breakdown of white matter tracts. Further research should therefore be undertaken to investigate the nature of causality, be it in the form of longitudinal, Mendelian Randomisation or experimental studies. Especially the latter is needed to further investigate the biological foundations and consequences of prolonged sleep as there are only limited hypotheses on its physiologic effects.

## 5 Limitations

To summarise and elaborate on the limitations of this study, several factors need to be considered. First, the measurement of sleep health variables was subjective and somewhat imprecise, relying, for example, on a single question about sleep quality rather than comprehensive questionnaires such as the ISI or PSQI. However, recent validation of the UK Biobank sleep quality question against both ISI and PSQI scores and structured interview diagnoses showed excellent sensitivity (98% and 94%, respectively) and specificity (96% and 89%, respectively) for detecting insomnia disorder (Hammerschlag et al. 2017). In addition, our study benefited from a large sample size, which increases the likelihood of replicating any true insomnia effects, even with potentially lower sensitivity and specificity. It is likely that these aspects also apply to sleep health variables other than insomnia, as sleep duration, chronotype, and daytime sleepiness tend to be even easier to objectify.

In addition, this study is limited to correlational findings, and no causal relationships can be inferred. We did not compare data over time or use advanced statistical techniques such as Mendelian randomisation or mediation/moderation analyses, which would have helped to explore potential causal mechanisms more rigorously.

Another limitation is the limited information provided by the axial diffusivity (AD) and radial diffusivity (RD) values. The inclusion of these variables, together with the application of a strict Bonferroni correction, reduces the alpha level and therefore the statistical power, potentially limiting our findings.

In addition, the study included only basic control for psychiatric conditions, with psychiatric medication and depression included as predictors and rigorously excluded in a sensitivity analysis. Given the possibility that psychiatric conditions may influence different subtypes of sleep health variables, more thorough control for these factors, particularly the inclusion of anxiety, is essential in future studies and may help to further differentiate effects on white matter integrity.

Finally, the UK Biobank sample is older than the general population, which may limit the generalisability of the findings. Sleep habits may have been established over a longer period of time, and brain ageing processes may be underway, making it unclear how applicable these results are to younger populations. Similarly, the UK Biobank cohort is known to be healthier than the general population. In our case, and as noted previously (Arora et al. 2023), the extremely low number of participants reporting sleep apnea in this sample (*n* = 155) seems implausible and is likely to be a gross underestimate.

## 6 Conclusion

In conclusion, this study suggests a hitherto unobserved correlation between affected white matter and long sleep duration. To a smaller extent, reduced white matter integrity was associated with daytime sleepiness and evening chronotype. White matter changes mainly presented as reduced FA and ICVF, as well as increased AD, RD, MD and ISOVF throughout wide‐spanning projection fibres of both hemispheres. Moreover, the data do not support the previous notion of altered white matter integrity in insomnia patients, specifically in the anterior internal capsule.

## Author Contributions

**Roxana Petri:** conceptualization, investigation, writing – original draft, methodology, visualization, writing – review and editing, formal analysis, data curation. **Florian Holub:** writing – review and editing. **Julian E. Schiel:** writing – review and editing, formal analysis. **Bernd Feige:** formal analysis. **Martin K. Rutter:** writing – review and editing, project administration, data curation. **Sandra Tamm:** writing – review and editing. **Dieter Riemann:** writing – review and editing. **Simon D. Kyle:** writing – review and editing. **Kai Spiegelhalder:** conceptualization, methodology, writing – review and editing, project administration, supervision, formal analysis, data curation.

## Conflicts of Interest

Martin K. Rutter has received non‐promotional speaker fees from Novo Nordisk, consultancy fees from Cell Catapult and Roche Diabetes Care, and modest ownership of shares in GlaxoSmithKline, outside the submitted work. Simon D. Kyle is supported by the National Institute for Health Research, Oxford Biomedical Research Centre based at Oxford, Oxford University Hospitals NHS Trust, and the University of Oxford. All other authors report no biomedical financial interests or potential conflicts of interest.

## Supporting information


Figure S1.

Figure S2.

Figure S3.

Figure S4.

Figure S5.

Figure S6.

**Data S1.** Supporting Information.

## Data Availability

The data that support the findings of this study are available from UK Biobank. Restrictions apply to the availability of these data, which were used under license for this study. Data are available from https://www.ukbiobank.ac.uk/ with the permission of UK Biobank.
