# Supplementary figures and images for: Sleep Health and White Matter Integrity in the UK Biobank
Source: J Sleep Res. 2025 Mar 12;34(6):e70034. doi: 10.1111/jsr.70034 (PMC12592828; doi:10.1111/jsr.70034)

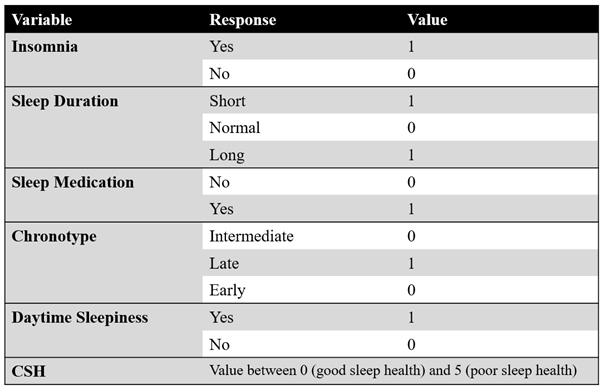

Supplement: Supplementary file 1 — Figure S1. [file JSR-34-e70034-s003.tiff]

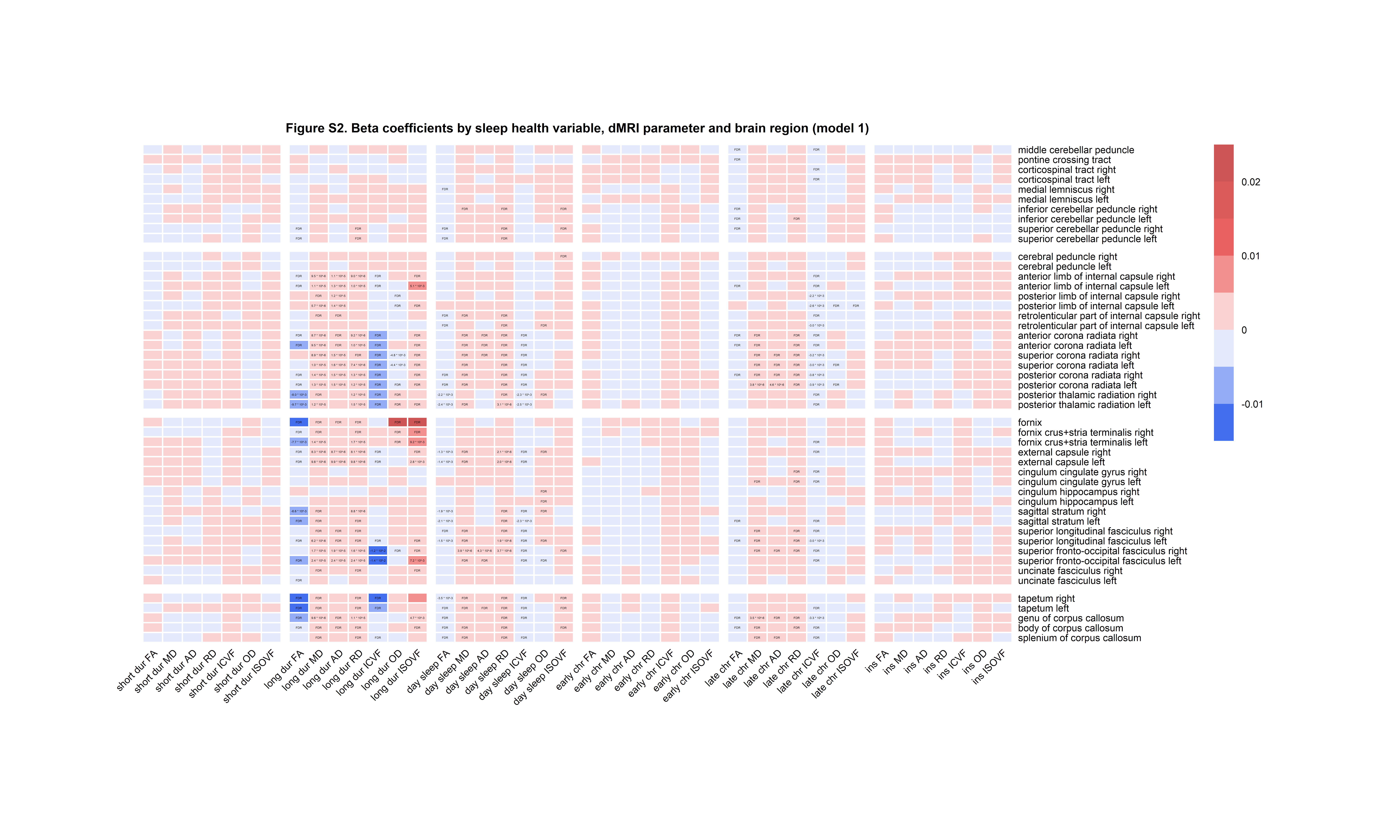

Supplement: Supplementary file 2 — Figure S2. [file JSR-34-e70034-s001.tiff]

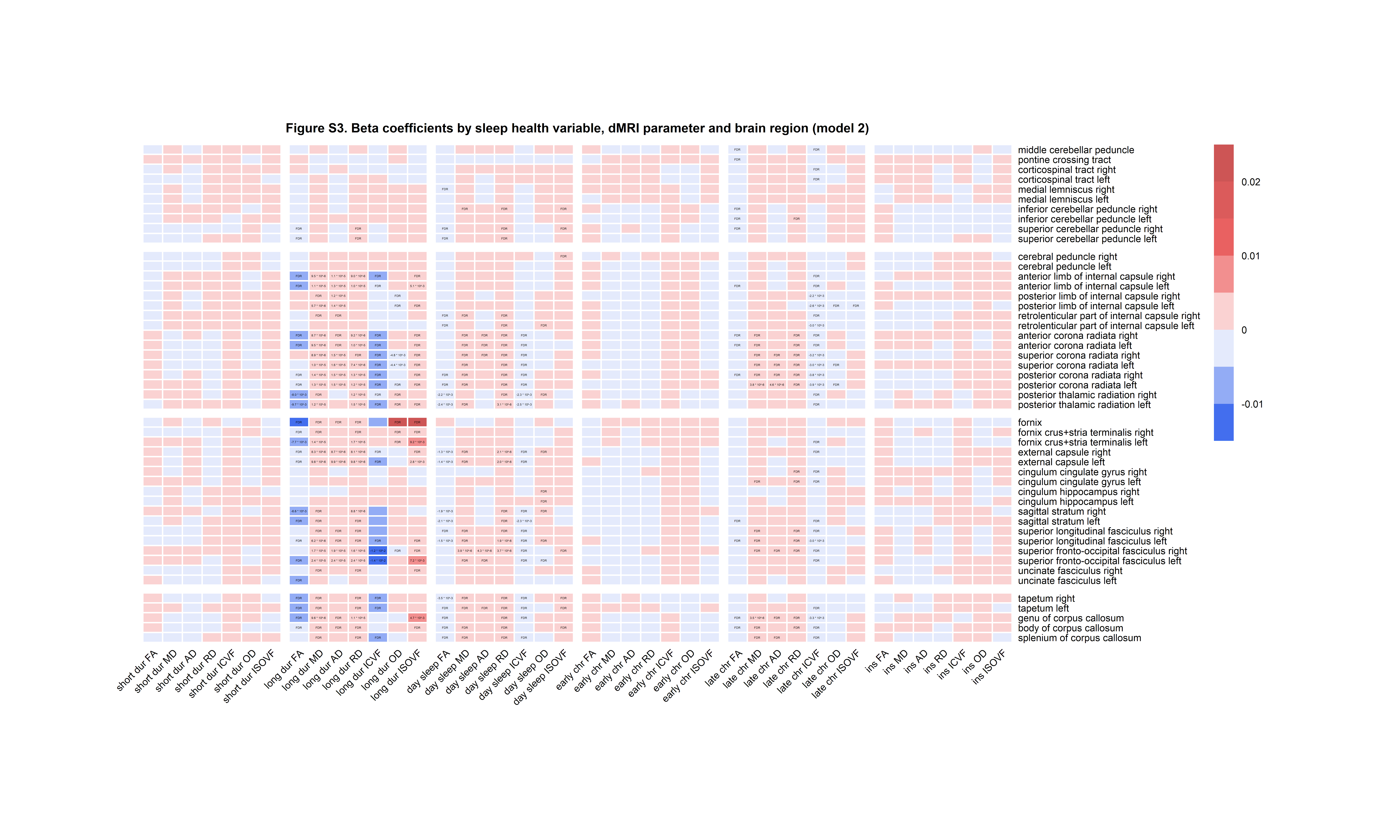

Supplement: Supplementary file 3 — Figure S3. [file JSR-34-e70034-s005.tiff]

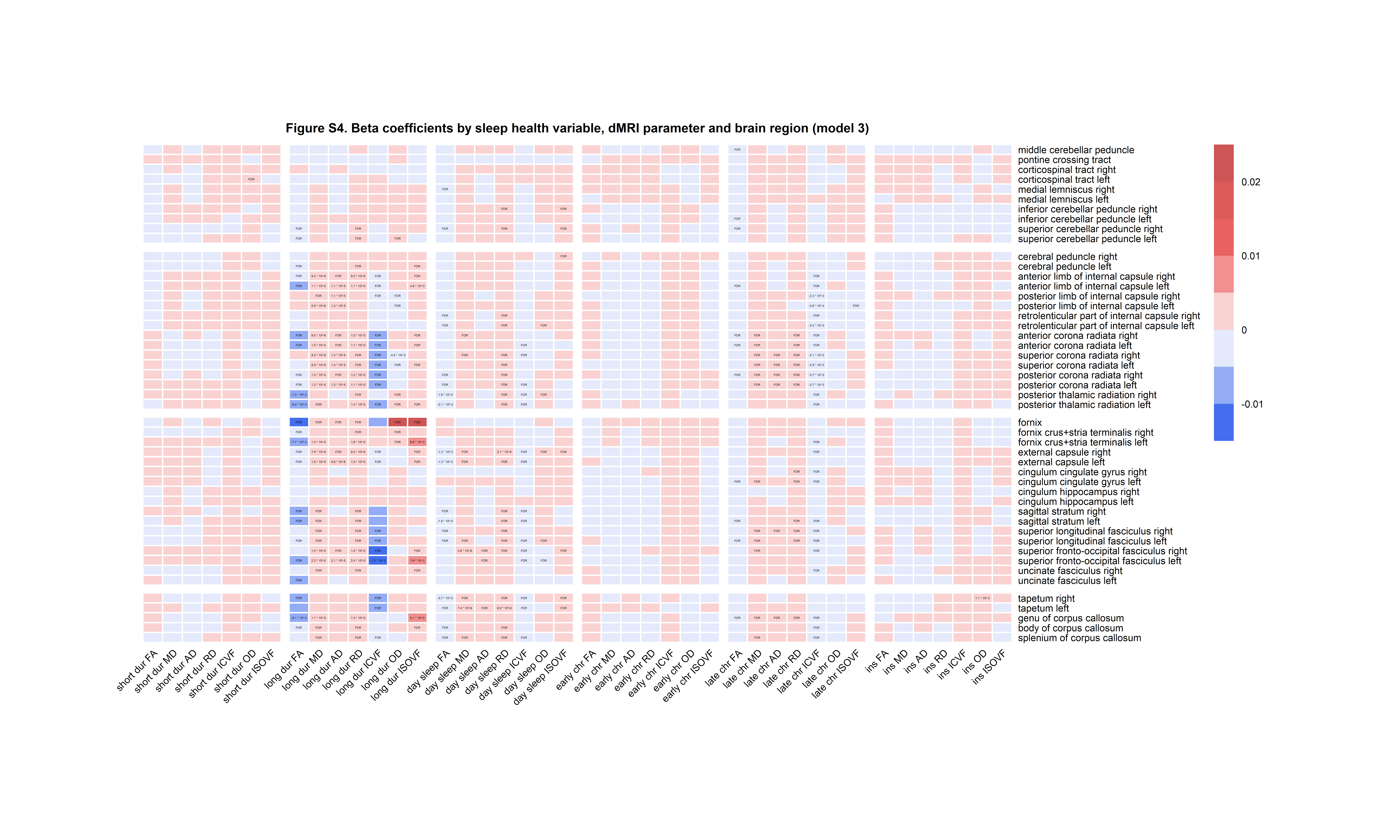

Supplement: Supplementary file 4 — Figure S4. [file JSR-34-e70034-s002.tiff]

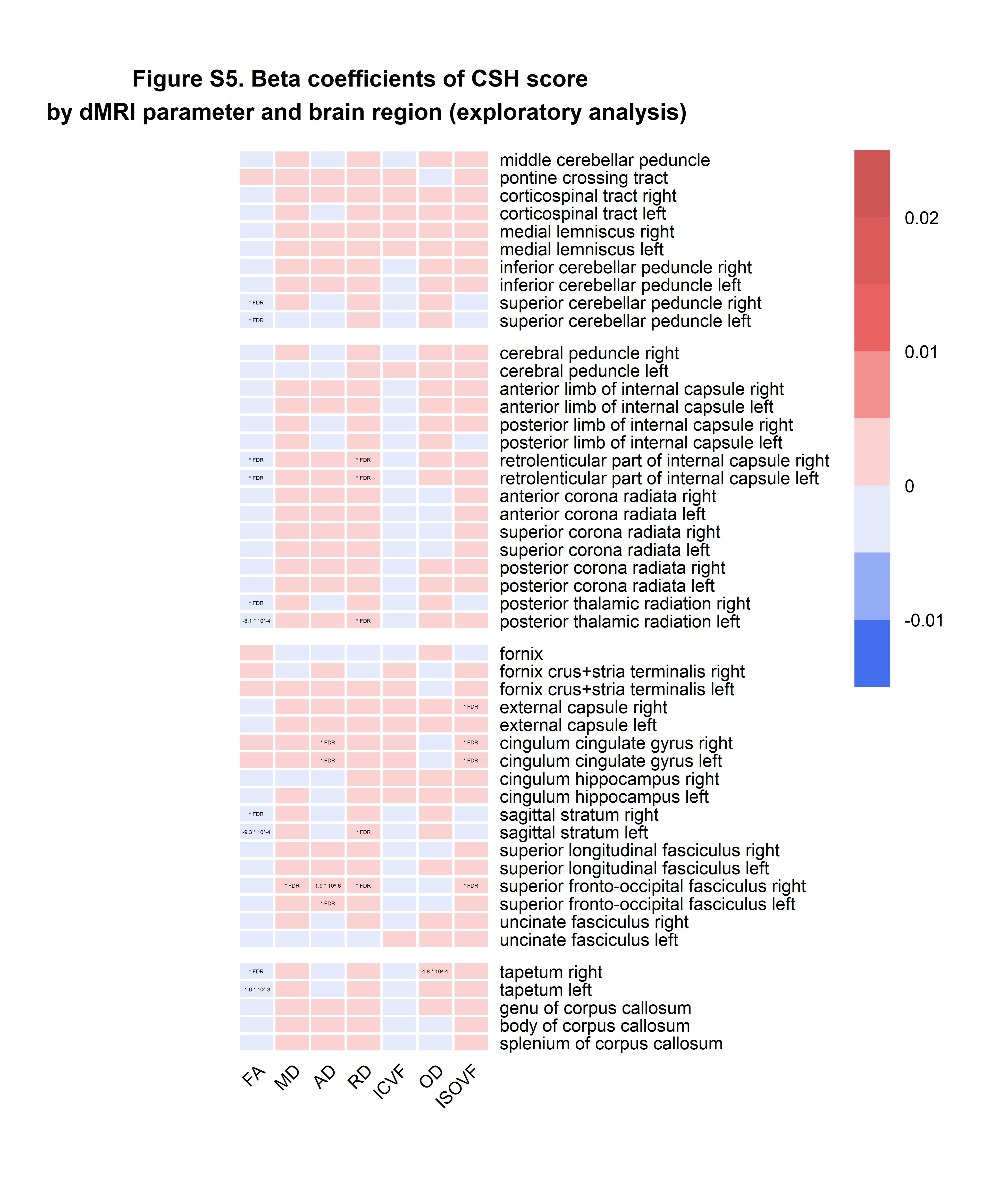

Supplement: Supplementary file 5 — Figure S5. [file JSR-34-e70034-s004.tiff]

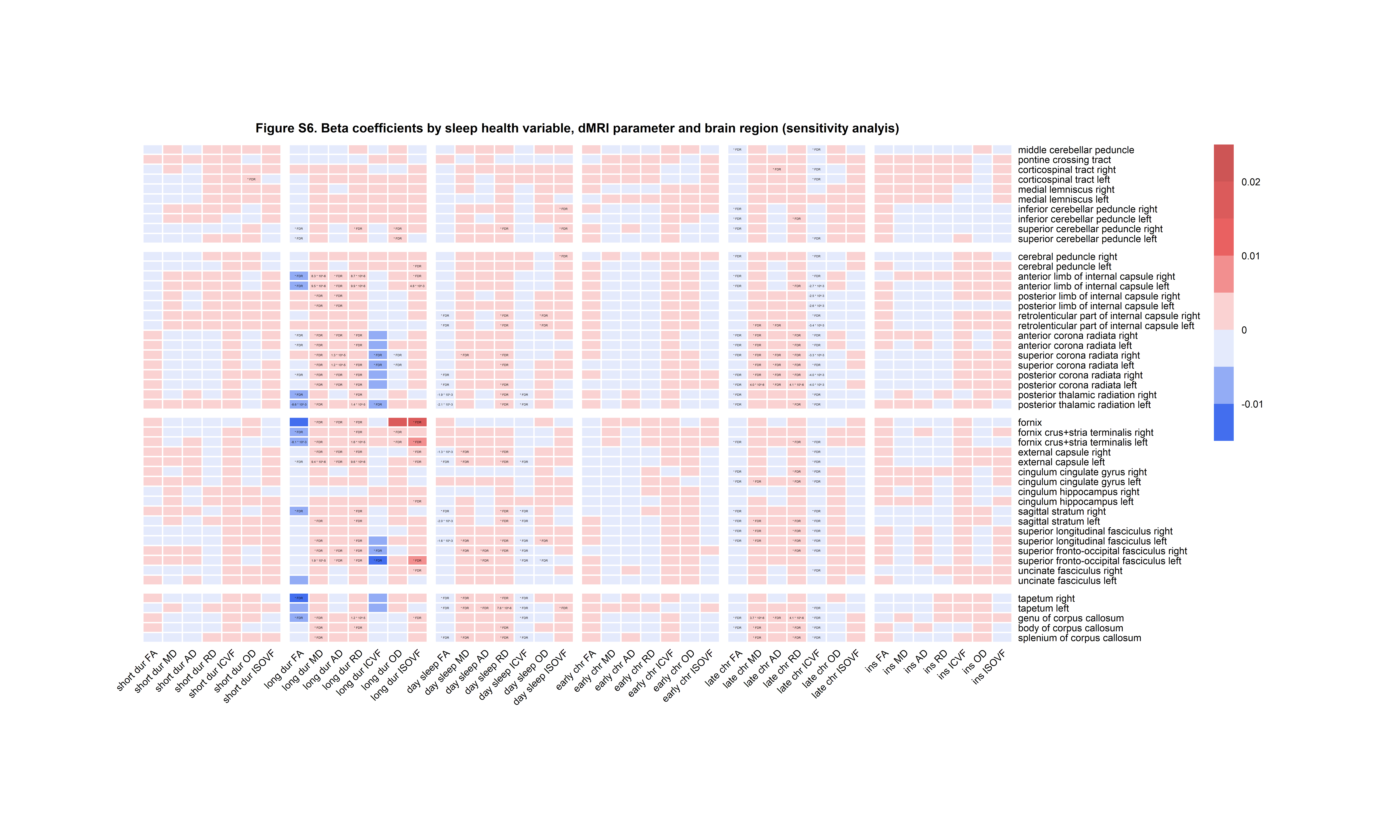

Supplement: Supplementary file 6 — Figure S6. [file JSR-34-e70034-s006.tiff]
