# Supplementary material for: Sleep Health and White Matter Integrity in the UK Biobank
Source: J Sleep Res. 2025 Mar 12;34(6):e70034. doi: 10.1111/jsr.70034 (PMC12592828; doi:10.1111/jsr.70034)
Supplement: Supplementary file 7 — Data S1. Supporting Information. [file JSR-34-e70034-s007.docx]

**Supplementary method section**

Diffusion MRI is sensitive to water diffusion, which is higher parallel than perpendicular to white matter axons. The degree and orientation of this anisotropy can be estimated by diffusion tensor imaging (DTI) ^1^. Parameters derived from the diffusion tensor model include fractional anisotropy (FA; directional coherence of water diffusion), mean diffusivity (MD; magnitude of overall diffusivity), axial diffusivity (AD; magnitude of diffusion along the principal axis of diffusion) and radial diffusivity (RD; magnitude of diffusion perpendicular to the principal axis of diffusion). These DTI indices are influenced by a variety of white matter properties such as axonal membrane integrity, degree of myelination and the extent of coherent alignment of fibres ^2,3^. Estimates of the relatively new *neurite orientation dispersion and density imaging* (NODDI) model ^4^ have shown to be of added value concerning the neurobiological interpretation of DTI parameters ^5^. By modelling different compartments of brain tissue (intra-cellular, extra-cellular and cerebrospinal fluid) with their respective diffusion properties, it provides further insights into white matter microstructure. Outputs of the NODDI model are estimates of neurite density (intra-cellular volume fraction; ICVF), tract disorganisation (orientation dispersion; OD) and proportion of extra-cellular water diffusion (isotropic volume fraction; ISOVF).

Figure S1. Computation of composite sleep health score (CSH) for the exploratory analysis


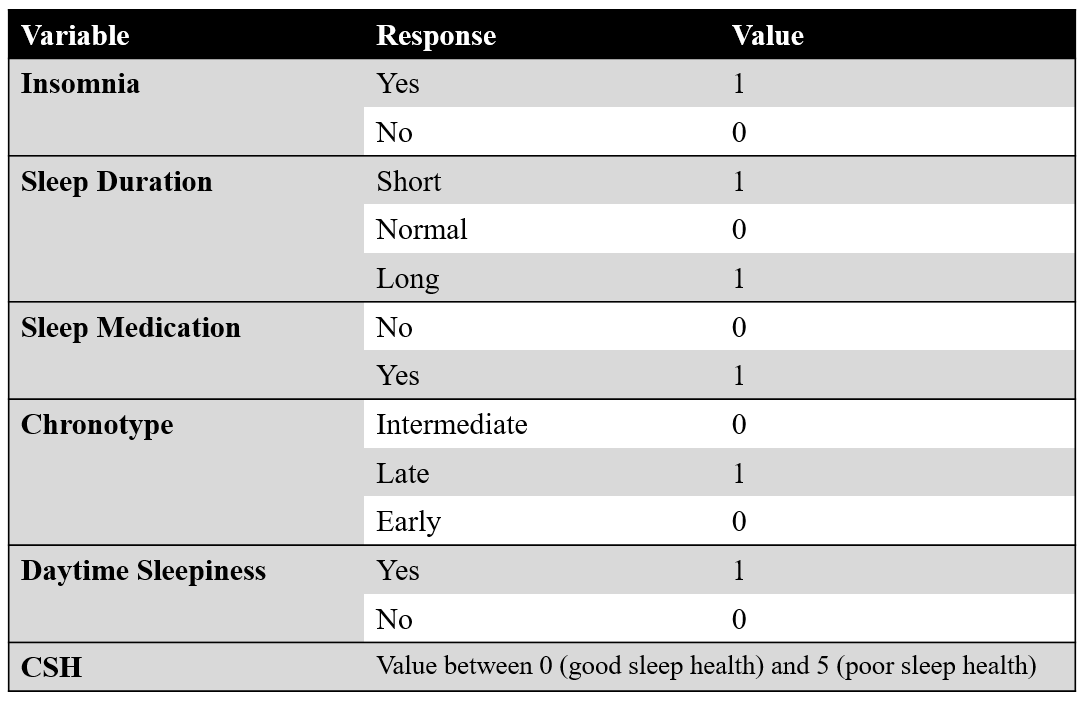


Figure S2. *Beta weights by sleep health dimension, dMRI parameter and brain region (model 1)*

^The magnitude of the beta weight can be extracted from its color, significant correlations contain the exact beta weight, correlations that were significant after FDR correction but not after Bonferroni correction are marked as “FDR”. The x-axis displays the sleep health variables (short dur = short sleep duration; long dur = long sleep duration; day sleep = daytime sleepiness; early chr = early chronotype; late chr = late chronotype; ins = insomnia symptoms) along with the dMRI parameters (FA = fractional anisotropy; MD = mean diffusivity; AD = axial diffusivity; RD = radial diffusivity; ICVF = intra-cellular volume fraction, OD = oriental dispersion; ISOVF = isotropic volume fraction), brain regions are listed on the y-axis and sorted into four blocks containing brain stem, projection, association and commissural tracts from top to bottom.^

Uploaded as separate tiff-file

Figure S3. *Beta weights by sleep health dimension, dMRI parameter and brain region (model 2)*

^The magnitude of the beta weight can be extracted from its color, significant correlations contain the exact beta weight, correlations that were significant after FDR correction but not after Bonferroni correction are marked as “FDR”.The x-axis displays the sleep health variables (short dur = short sleep duration; long dur = long sleep duration; day sleep = daytime sleepiness; early chr = early chronotype; late chr = late chronotype; ins = insomnia symptoms) along with the dMRI parameters (FA = fractional anisotropy; MD = mean diffusivity; AD = axial diffusivity; RD = radial diffusivity; ICVF = intra-cellular volume fraction, OD = oriental dispersion; ISOVF = isotropic volume fraction), brain regions are listed on the y-axis and sorted into four blocks containing brain stem, projection, association and commissural tracts from top to bottom.^

Uploaded as separate tiff-file

Figure S4. *Beta weights by sleep health dimension, dMRI parameter and brain region (model 3)*

^The magnitude of the beta weight can be extracted from its color, significant correlations contain the exact beta weight, correlations that were significant after FDR correction but not after Bonferroni correction are marked as “FDR”. The x-axis displays the sleep health variables (short dur = short sleep duration; long dur = long sleep duration; day sleep = daytime sleepiness; early chr = early chronotype; late chr = late chronotype; ins = insomnia symptoms) along with the dMRI parameters (FA = fractional anisotropy; MD = mean diffusivity; AD = axial diffusivity; RD = radial diffusivity; ICVF = intra-cellular volume fraction, OD = oriental dispersion; ISOVF = isotropic volume fraction), brain regions are listed on the y-axis and sorted into four blocks containing brain stem, projection, association and commissural tracts from top to bottom.^

Uploaded as separate tiff-file

Figure S5. *Beta weights of composite sleep health by dMRI parameter and brain region (exploratory analysis)*

^The magnitude of the beta weight can be extracted from its color, significant correlations contain the exact beta weight, correlations that were significant after FDR correction but not after Bonferroni correction are marked as “FDR”. The x-axis displays the dMRI parameters (FA = fractional anisotropy; MD = mean diffusivity; AD = axial diffusivity; RD = radial diffusivity; ICVF = intra-cellular volume fraction; OD = oriental dispersion; ISOVF = isotropic volume fraction), brain regions are listed on the y-axis and sorted into four blocks containing brain stem, projection, association and commissural tracts from top to bottom.^

Uploaded as separate tiff-file

Figure S6. *Beta weights by sleep health dimension, dMRI parameter and brain region (sensitivity analysis)*

^The magnitude of the beta weight can be extracted from its color, significant correlations contain the exact beta weight, correlations that were significant after FDR correction but not after Bonferroni correction are marked as “FDR”. The x-axis displays the sleep health variables (short dur = short sleep duration; long dur = long sleep duration; day sleep = daytime sleepiness; early chr = early chronotype; late chr = late chronotype; ins = insomnia symptoms) along with the dMRI parameters (FA = fractional anisotropy; MD = mean diffusivity; AD = axial diffusivity; RD = radial diffusivity; ICVF = intra-cellular volume fraction, OD = oriental dispersion; ISOVF = isotropic volume fraction), brain regions are listed on the y-axis and sorted into four blocks containing brain stem, projection, association and commissural tracts from top to bottom.^

Uploaded as separate tiff-file

Table S1. Sample size and exclusion

| Sample with complete DTI/NODDI measures | 37,441/37,439 |
| --- | --- |
| Exclusion because of neurological disease | 894 |
| Exclusion because of sleep apnoea | 155 |
| Exclusion because of incomplete sleep variables | 3,530 |
| Exclusion because of incomplete sex, age, BMI or brain volume | 1,092 |
| Remaining sample size for linear model 1 (DTI/NODDI) | 31,770/31,769 |
| Exclusion because of incomplete education, TDI or depressive symptoms | 2,655 |
| Remaining sample size for linear model 2 + 3 (DTI/NODDI) + exploratory analysis | 29,115/29,114 |
| Exclusion because of F-diagnosis (psychiatric disorders) | 1,504 |
| Exclusion because of G-diagnosis (neurological disorders) | 1,608 |
| Remaining sample size for sensitivity analysis | 26,003 |

Table S2. *Sleep medication*

| **Biobank code** | **Sleep medication** |
| --- | --- |
| 1140863152 | diazepam |
| 1141157496 | diazepam product |
| 1140863244 | valium 2mg tablet |
| 1140863250 | valium 2mg/5ml syrup |
| 1140855856 | valium 10mg suppository |
| 1140863202 | temazepam |
| 1140863210 | normison 10mg capsule |
| 1140863138 | euhypnos 10mg/5ml oral solution |
| 1140863144 | zopiclone |
| 1140928004 | zimovane ls 3.75mg tablet |
| 1141171404 | zaleplon |
| 1141171410 | sonata 5mg capsule |
| 1140865016 | zolpidem |
| 1140864916 | stilnoct 5mg tablet |
| 1140863182 | nitrazepam |
| 1140863194 | mogadon 5mg tablet |
| 1140855896 | nitrados 5mg tablet |
| 1140863196 | remnos 5mg tablet |
| 1140855900 | somnite 5mg tablet |
| 1140855898 | noctesed 5mg tablet |
| 1140855902 | surem 5mg capsule |
| 1140855904 | unisomnia 5mg tablet |
| 1140863104 | flunitrazepam |
| 1140863106 | rohypnol 1mg tablet |
| 1140855914 | triazolam |
| 1140855920 | halcion 125micrograms tablet |

Table S3. *Psychotropic medication*

| **Biobank code** | **Psychotropic medication** |  |
| --- | --- | --- |
| 1140867490 | lithium product | |
| 1140867504 | priadel 200mg m/r tablet | |
| 1140867494 | camcolit 250 tablet | |
| 1140872198 | sodium valproate | |
| 1140872200 | epilim 100mg crushable tablet | |
| 1141172838 | depakote 250mg e/c tablet | |
| 1140872214 | valproic acid | |
| 1140872064 | carbamazepine product | |
| 2038459704 | carbamazepine | |
| 1140872072 | tegretol 100mg tablet | |
| 1141167860 | teril cr 200mg m/r tablet | |
| 1141185460 | teril retard 200mg m/r tablet | |
| 1141162898 | timonil retard 200mg m/r tablet | |
| 1140864452 | epimaz 100mg tablet | |
| 1140867888 | paroxetine |  |
| 1140882236 | seroxat 20mg tablet | |
| 1140879540 | fluoxetine |  |
| 1140867876 | prozac 20mg capsule | |
| 1140921600 | citalopram |  |
| 1141151946 | cipramil 10mg tablet | |
| 1141180212 | escitalopram | |
| 1141190158 | cipralex 5mg tablet | |
| 1140867878 | sertraline |  |
| 1140867884 | lustral 50mg tablet | |
| 1140879544 | fluvoxamine | |
| 1141152732 | mirtazapine |  |
| 1141152736 | zispin 30mg tablet | |
| 1141200564 | duloxetine |  |
| 1141201834 | cymbalta 30mg gastro-resistant capsule | |
| 1141200570 | yentreve 20mg gastro-resistant capsule | |
| 1140916282 | venlafaxine |  |
| 1140916288 | efexor 37.5mg tablet | |
| 1140879616 | amitriptyline | |
| 1140867658 | elavil 10mg tablet | |
| 1140867668 | tryptizol 10mg tablet | |
| 1140867662 | lentizol 25mg m/r capsule | |
| 1140867948 | amitriptyline hydrochloride+perphenazine 10mg/2mg tablet | |
| 1140867934 | triptafen tablet | |
| 1140867938 | amitriptyline+chlordiazepoxide 12.5mg/5mg capsule | |
| 1140856186 | limbitrol 10 capsule | |
| 1140867928 | limbitrol-5 capsule | |
| 1140867850 | phenelzine |  |
| 1140910704 | maoi - phenelzine | |
| 1140867852 | nardil 15mg tablet | |
| 1140867920 | moclobemide | |
| 1140867922 | manerix 150mg tablet | |
| 1140879630 | imipramine |  |
| 1140867712 | tofranil 10mg tablet | |
| 1140867756 | trimipramine | |
| 1140867758 | surmontil 10mg tablet | |
| 1140879628 | dothiepin |  |
| 1140909806 | dosulepin |  |
| 1140867624 | prothiaden 25mg capsule | |
| 1141171824 | thaden 25mg capsule | |
| 1140879620 | clomipramine | |
| 1140867690 | anafranil 10mg capsule | |
| 1140867726 | lofepramine |  |
| 1140882310 | gamanil 70mg tablet | |
| 1141146062 | lomont 70mg/5ml s/f suspension | |
| 1140879556 | mianserin |  |
| 1140867806 | bolvidon 10mg tablet | |
| 1140867812 | norval 10mg tablet | |
| 1140879658 | chlorpromazine | |
| 1140910358 | cpz - chlorpromazine | |
| 1140863416 | largactil 10mg tablet | |
| 1140867168 | haloperidol |  |
| 1140867184 | haldol 5mg tablet | |
| 1140867092 | serenace 500micrograms capsule | |
| 1140867398 | fluphenazine decanoate | |
| 1140882098 | fluphenazine | |
| 1140867456 | modecate 12.5mg/0.5ml oily injection | |
| 1140867156 | moditen 1mg tablet | |
| 1140856004 | moditen enanthate 25mg/ml injection | |
| 1140909800 | flupentixol |  |
| 1140867150 | flupenthixol |  |
| 1140867152 | depixol 3mg tablet | |
| 1140867952 | fluanxol 500micrograms tablet | |
| 1140882100 | zuclopenthixol | |
| 1140867342 | clopixol 2mg tablet | |
| 1140867406 | loxapine |  |
| 1140867414 | loxapac 10mg capsule | |
| 1140867084 | droperidol |  |
| 1140867086 | droleptan 10mg tablet | |
| 1140868120 | trifluoperazine | |
| 1140867244 | stelazine 1mg tablet | |
| 1140879750 | thioridazine |  |
| 1140867312 | melleril 10mg tablet | |
| 1141152848 | quetiapine |  |
| 1141152860 | seroquel 25mg tablet | |
| 1140867444 | risperidone |  |
| 1141177762 | risperdal 0.5mg tablet | |
| 1140928916 | olanzapine |  |
| 1141167976 | zyprexa 2.5mg tablet | |
| 1141195974 | aripiprazole |  |
| 1141202024 | abilify 5mg tablet | |
| 1141153490 | amisulpride |  |
| 1141184742 | solian 100mg/ml s/f oral solution | |
| 1140867420 | clozapine |  |
| 1140882320 | clozaril 25mg tablet | |

Table S4. *Antihypertensive medication*

| **Biobank code** | **Antihypertensive medication** |
| --- | --- |
| 1140860750 | Captopril |
| 1140860764 | captopril+hydrochlorothiazide 25mg/12.5mg tablet |
| 1140860790 | enalaprilmaleate+hydrochlorothiazide 20mg/12.5mg tablet |
| 1140888552 | Enalapril |
| 1140888556 | Fosinopril |
| 1140860696 | Lisinopril |
| 1140864952 | lisinopril+hydrochlorothiazide 10mg/12.5mg tablet |
| 1140888560 | perindopril |
| 1141180592 | perindopril+indapamide |
| 1140860728 | quinapril |
| 1140860806 | ramipril |
| 1141165470 | felodipine+ramipril |
| 1140860904 | trandolapril |
| 1141153328 | trandolapril+verapamil hydrochloride |
| 1141156836 | candesartan cilexetil |
| 1141171336 | eprosartan |
| 1141172682 | irbesartan+hydrochlorothiazide 150mg/12.5mg tablet |
| 1141152998 | irbesartan |
| 1140916356 | losartan |
| 1141151016 | losartan potassium+hydrochlorothiazide 50mg/12.5mg tablet |
| 1141193282 | olmesartan |
| 1141166006 | telmisartan |
| 1141187788 | telmisartan+hydrochlorothiazide 40mg/12.5mg tablet |
| 1141201038 | valsartan+hydrochlorothiazide 80mg/12.5mg tablet |
| 1141145660 | valsartan |
| 1140909368 | carvedilol |
| 1140866738 | atenolol |
| 1141146124 | atenolol+chlorthalidone |
| 1141146126 | atenolol+bendrofluazide |
| 1141146128 | atenolol+co-amilozide |
| 1141180778 | atenolol+chlortalidone |
| 1141194810 | atenolol+bendroflumethiazide |
| 1140860426 | atenolol+nifedipine 50mg/20mg m/r capsule |
| 1140879760 | bisoprolol |
| 1140864950 | bisoprolol fumarate+hydrochlorothiazide 10mg/6.25mg tablet |
| 1140879818 | metoprolol |
| 1140860308 | metoprolol tartrate+chlorthalidone 100mg/12.5mg tablet |
| 1140860404 | metoprolol tartrate+hydrochlorothiazide 100mg/12.5mg tablet |
| 1141164276 | nebivolol |
| 1140879806 | diltiazem |
| 1140926778 | diltiazem hcl+hydrochlorothiazide 150mg/12.5mg m/r capsule |
| 1140879802 | amlodipine |
| 1140888646 | felodipine |
| 1140861276 | lacidipine |
| 1141153026 | lercanidipine |
| 1140861088 | nifedipine |
| 1140888510 | verapamil |
| 1140888512 | amiloride |
| 1140866422 | amiloride hcl+cyclopenthiazide 2.5mg/250micrograms tablet |
| 1140866426 | amiloride hydrochloride+bumetanide 5mg/1mg tablet |
| 1140866236 | spironolactone |
| 1140866280 | bumetanide |
| 1140866448 | bumetanide+potassium 500micrograms/7.7mmol m/r tablet |
| 1140909706 | chlortalidone |
| 1141180772 | triamterene+chlortalidone 50mg/50mg tablet |
| 1141195254 | triamterene+furosemide 50mg/40mg tablet |
| 1141195258 | furosemide+potassium 20mg/10mmol m/r tablet |
| 1140909708 | furosemide |
| 1140866078 | indapamide |
| 1141194794 | bendroflumethiazide |
| 1141194800 | bendroflumethiazide+potassium 2.5mg/7.7mmol m/r tablet |
| 1141194804 | nadolol+bendroflumethiazide 40mg/5mg tablet |
| 1141194808 | timolol maleate+bendroflumethiazide 10mg/2.5mg tablet |
| 1140860332 | sotalol hydrochloride+hydrochlorothiazide 80mg/12.5mg tablet |
| 1140860422 | acebutolol+hydrochlorothiazide 200mg/12.5mg tablet |
| 1140860562 | methyldopa+hydrochlorothiazide 250mg/15mg tablet |
| 1140866162 | hydrochlorothiazide |
| 1140883524 | cinnarizine |
| 1140871986 | clonidine hydrochloride 25micrograms tablet |
| 1140883468 | clonidine |
| 1140928284 | moxonidine |
| 1140879778 | doxazosin |
| 1140879774 | alfuzosin |
| 1140926934 | tamsulosin |
| 1140879798 | terazosin |
| 1140879826 | levobunolol |
| 1140860418 | propranolol hydrochloride+bendrofluazide 80mg/2.5mg capsule |
| 1140879842 | propranolol |
| 1140879854 | sotalol |
| 1140860336 | timolol maleate+co-amilozide 10mg/2.5mg/25mg tablet |
| 1140860340 | timolol maleate+bendrofluazide 10mg/2.5mg tablet |
| 1140860342 | timolol maleate+bendrofluazide 20mg/5mg tablet |
| 1140875840 | timolol 0.25% eye drops |
| 1140879866 | timolol |
| 1141169516 | dorzolamide+timolol |
| 1141184722 | latanoprost+timolol |

Table S5. *Cardiovascular diseases that were used as exclusion criteria in the current study*

| **Biobank code** | **Cardiovascular disease** |
| --- | --- |
| 1065 | Hypertension |
| 1067 | peripheral vascular disease |
| 1068 | venous thromboembolic disease |
| 1072 | essential hypertension |
| 1073 | gestational hypertension/pre-eclampsia |
| 1074 | Angina |
| 1075 | heart attack/myocardial infarction |
| 1076 | heart failure/pulmonary oedema |
| 1077 | heart arrhythmia |
| 1078 | heart valve problem/heart murmur |
| 1079 | Cardiomyopathy |
| 1080 | pericardial problem |
| 1081 | Stroke |
| 1082 | transient ischaemic attack (tia) |
| 1083 | subdural haemorrhage/haematoma |
| 1086 | subarachnoid haemorrhage |
| 1087 | leg claudication/ intermittent claudication |
| 1088 | arterial embolism |
| 1093 | pulmonary embolism +/- dvt |
| 1094 | deep venous thrombosis (dvt) |

Table S6. *Neurological diseases that were used as exclusion criteria in the current study*

| **Biobank code** | **Neurological Disease** |
| --- | --- |
| 1491 | brain haemorrhage |
| 1245 | brain abscess/intracranial abscess |
| 1425 | cerebral aneurysm |
| 1433 | cerebral palsy |
| 1258 | chronic/degenerative neurological problem |
| 1263 | dementia/alzheimers/cognitive impairment |
| 1246 | encephalitis |
| 1264 | epilepsy |
| 1266 | head injury |
| 1244 | infection of nervous system |
| 1583 | ischaemic stroke |
| 1659 | meningioma / benign meningeal tumour |
| 1247 | meningitis |
| 1259 | motor neurone disease |
| 1261 | multiple sclerosis |
| 1240 | neurological injury/trauma |
| 1683 | benign neuroma |
| 1397 | other demyelinating disease (not multiple sclerosis) |
| 1434 | other neurological problem |
| 1262 | parkinsons disease |
| 1524 | spina bifida |
| 1086 | subarachnoid haemorrhage |
| 1083 | subdural haemorrhage/haematoma |
| 1082 | transient ischaemic attack (tia) |

Table S7. *Amount of significant white matter tracts according to model and type of correction*

| Model | Correction | Insom-nia | Short Duration | Long Duration | Early Chronotype | Late  Chronotype | Daytime  Sleepiness | CSH |
| --- | --- | --- | --- | --- | --- | --- | --- | --- |
| 1 | Bonf. | 0 | 0 | 58 | 0 | 12 | 18 | NA |
|  | FDR | 0 | 0 | 153 | 0 | 81 | 105 | NA |
| 2 | Bonf. | 0 | 0 | 50 | 0 | 5 | 14 | NA |
|  | FDR | 0 | 1 | 147 | 0 | 66 | 90 | NA |
| 3 | Bonf. | 1 | 0 | 46 | 0 | 7 | 10 | NA |
|  | FDR | 1 | 1 | 137 | 0 | 68 | 78 | NA |
| Sensiti-vity | Bonf. | 0 | 0 | 15 | 0 | 11 | 6 | NA |
|  | FDR | 0 | 1 | 95 | 0 | 97 | 63 | NA |
| Explora-tory | Bonf. | NA | NA | NA | NA | NA | NA | 5 |
|  | FDR | NA | NA | NA | NA | NA | NA | 25 |

# **References**

1 Basser PJ, Mattiello J, Lebihan D. Estimation of the Effective Self-Diffusion Tensor from the NMR Spin Echo. *J Magn Reson B* 1994; **103**: 247–254.

2 Beaulieu C. The basis of anisotropic water diffusion in the nervous system – a technical review. *NMR Biomed* 2002; : 21.

3 Tournier J-D, Mori S, Leemans A. Diffusion Tensor Imaging and Beyond. *Magn Reson Med* 2011; **65**: 1532–1556.

4 Zhang H, Schneider T, Wheeler-Kingshott CA, Alexander DC. NODDI: Practical in vivo neurite orientation dispersion and density imaging of the human brain. *NeuroImage* 2012; **61**: 1000–1016.

5 Timmers I, Roebroeck A, Bastiani M, Jansma B, Rubio-Gozalbo E, Zhang H. Assessing Microstructural Substrates of White Matter Abnormalities: A Comparative Study Using DTI and NODDI. *PLOS ONE* 2016; **11**: e0167884.
